# Dietary supplementation with different types of fiber in gestation and lactation: effects on sow serum biochemical values and performance

**DOI:** 10.5713/ajas.19.0545

**Published:** 2019-10-21

**Authors:** Ruey-Chee Weng

**Affiliations:** 1 Department of Animal Science, National Pingtung University of Science and Technology, Neipu Pingtung 91201, Taiwan

**Keywords:** Dietary Fiber, Gestation, Lactation, Sow and Litter Performance, Serum Biochemical Values

## Abstract

**Objective:**

Three types of dietary fiber were fed to sows during gestation and lactation stages to monitor their physiological and metabolic adaptations during the pre-partum period and to determine how these effects may influence the lactation period and sow performance.

**Methods:**

Soon after breeding, 54 sows were selected and were fed with 20% supplementation as fed of wheat bran (WB), soya hulls (SH), or rice hulls (RH) in diets during gestation and lactation. Sows were weighed, backfat thickness was measured ultrasonically and jugular blood samples were collected from all sows. The litter size was equalized to 10, by fostering piglets from sows on the same treatment.

**Results:**

Sows gained 22.0, 21.8, and 25.5 kg of net maternal body weight during gestation (for WB, SH, and RH sows, respectively; p = 0.007). There was no treatment effect on the body weight change during lactation (p = 0.158), however RH sows consumed an average of 133.66 kg of feed, WB sows took 121.29 kg and SH sows took 126.77 kg during lactation (p<0.001). The SH litters gained an average of 59.34 kg of weight during lactation, while other litters gained 51.58 and 49.98 kg (for WB and RH litters, respectively; p<0.001). Exception for aspartate aminotransferase and alanine aminotransferase, measured serum biochemical values were broadly in agreement with earlier reports. Despite the use of additional vegetable oil to balance the energy level, RH sows still had lower concentrations of serum triglycerides in late gestation.

**Conclusion:**

Different types of fibrous ingredients in the gestation diet influenced most of the investigated reference values for sows. The values of serum biochemical parameters were generally not affected by fiber type during the lactation stage. The SH supplementation for sows is an effective approach to give heavier litters at birth and weaning and to increase voluntary feed intake in early lactation.

## INTRODUCTION

Supplying high-fiber diets during pregnancy has been shown to improve the welfare of sows [1] and increase feed intake over the subsequent lactation period [2–4]. Recently, Meunier-Salaün and colleagues [5] summarized and concluded that provision of dietary fibers during gestation generally has a beneficial effect on the behavior and welfare of sows which are restricted-fed. Moreover, sows fed with a high-fiber diet during gestation can manage their own feed transition, choosing the lactation diet spontaneously in the week preceding parturition [6]. This indicates that a high-fiber gestation diet promotes early intake of the lactation diet. When energy intake per sow was equalized among the gestation treatments, a higher percentage of the sows fed a diet containing 50% alfalfa hay had an improved piglet survival rate [7]. However, the multiple functionalities of fiber also depend on the type of fiber, the animal’s gastric adaption and other additional requirements during metabolic processes. Inclusion of increased amounts of dietary fiber may reduce hunger of restrict-fed pregnant sows due to a reduced assimilation of energy derived from starch, at the expense of greater amounts of energy derived from short-chain fatty acids arising from microbial fermentation of non-starch polysaccharides in the large intestine [8,9].

As can be seen from the above studies, fiber does have significant effects on sow nutrition and welfare. Even though serum biochemistry has been used more frequently for routine diagnosis in humans and laboratory animals, it can also be used in domestic animals. Measuring serum biochemical parameters of farm animals can provide important information on health and metabolism [10] and is a practical diagnostic tool for assessing pathological conditions in the live animal or for monitoring the health status of groups of animals [11].

Most studies in pigs have focused on sows, and biochemical parameters are influenced by a number of different factors such as diet, stage of gestation and lactation [12] and disease [13]. However, the management, housing, feeding and genetic makeup of sows have changed dramatically in recent years. The high production levels of sows which are seen in current pig production significantly influence the health and metabolic status of the sows, and consequently have changed the serum biochemical values [11].

Altering traditional corn-soybean diets to incorporate high fiber ingredients [14,15] and modifying feeding management can improve performance of sows. Using local resources or agricultural by-products is an effective approach to reduce feed cost, as well as to minimize carbon-footprints of rations for livestock and poultry. Animal production in areas with limited grain production mostly relies on imported grains; thus, the search for locally-produced useful resources for livestock is a matter of urgency, particularly in the wake of high grain prices and increasing demand for reduction in global warming potential from agriculture. For this reason, despite possible negative impacts on performance, increasing attention has been paid to dietary fiber in monogastric animals’ nutrition due to its multiple functionalities, especially in sow diets.

Therefore, the purpose of this study was to investigate the effects of feeding sows with alternative agricultural by-products during gestation and lactation on their physiological and metabolic adaptations during the peripartum period, and to determine how these effects may carry over to the lactation period and relate to sow serum biochemical values and reproductive performance. It was hypothesized that, when energy intake per sow was equalized among the gestation treatments, soya hulls (SH) or rice hulls (RH) could replace wheat bran (WB) in sow diets without changing their physiological and metabolic adaptations.

## MATERIALS AND METHODS

### Animal care

The experiment was carried out at the agriculture college practical farm of the National Pingtung University of Science and Technology, Taiwan. All procedures were in accordance with the Animal Production Act for Care and Use of Agricultural Animals in Research (Amended Date: 2018-12-26) and approved by the Council of Agriculture, Taiwan.

### Experimental design and animal housing

From the time of weaning, sows (Large White×Landrace or Landrace×Large White) of 3rd or 4th parity were housed in group pens and fed with a commercial gestating sow diet. All sows were given daily boar contact for 15 minutes in the early morning and late afternoon. When signs of estrus were seen, the sows received two inseminations at 12 and 24 hours after a stable reaction to a “back pressure test”. The fresh semen was mixed from different Duroc boars and diluted to 50×10^8^ sperm per 100 mL dose. At 21 and 30 days after insemination, the ultrasonic pregnancy detector PREG-TONE (Renco Corporation Inc., Minneapolis, MN, USA) was used to test each sow for pregnancy. After insemination, all the experimental sows were moved to individual stalls and received experimental diets. After excluding conception failures, 54 gestating sows were used as experimental animals and allocated to one of three dietary treatments for the whole duration of the experiment. At 109 days after insemination, all gestating sows were moved to the farrowing house for 28 days of nursing, and then moved back to the previous dry sow accommodation after weaning. No hormone treatments were allowed for induction of parturition. The litter size was equalized to 10 piglets by cross-fostering at 2 to 3 days old from sows on the same treatment; other sows were made available to take surplus piglets if necessary.

Starting at 2 days after insemination, the selected sows were fed with diets containing a 20% as fed supplementation of WB, SH, or RH during gestation and lactation. The composition and chemical characteristics of the experimental diets are described in Table 1.

After mating, the sows were kept in stalls and were fed twice a day at a level to supply an equal intake of metabolisable energy. Five days before the expected farrowing date (based on 114 day gestation), the sows were moved to the farrowing house. The diet was reduced to 2.0 kg per day per sow and experimental diets continued to be fed. Any sows that did not give birth by 114 days of pregnancy continued to be fed 2.0 kg per day. Any uneaten feed was cleaned out before the next feeding, and it was not returned to the experimental animals. All feed refusals were recorded on the data sheets, and corrected to dry matter (DM, determined by drying at 60°C). From the initial day of pregnancy until parturition, sows had no feed refusals. According to NRC standards [16] on energy requirements for pregnancy and lactation the daily feed allowances, and thus feed intakes, of WB, SH, and RH sows were 2.02, 2.05, and 2.10 kg per day of the experimental rations from 1 to 80 days of pregnancy, and 2.39, 2.42, and 2.48 kg per day of the experimental rations from 81 days of pregnancy until 110 days of pregnancy, respectively. The feed was increased postpartum by 0.5 kg DM per day till sows reached *ad libitum* intake. During lactation, the feed amount was divided into three meals per day. No creep feed was given to piglets. After weaning, the feed amount was reduced to 3 kg per sow per day and sows were fed twice a day until rebreeding. The health of the animals was checked daily and, if necessary, sick animals were treated by the veterinarian. The sow and piglets received Hog cholera and Swine erysipelas vaccination injections at 21 days post parturition. Piglets were not subjected to teeth clipping, tail docking or castration.

### Data collection

Sows were weighed and backfat thickness was measured ultrasonically (Preg-Alert Pro, Renco Corporation Inc., Minneapolis, MN, USA) at the last rib, 6.5 cm from the midline on each side, at mating, 30, 80, and 110 days after insemination and at weaning. Piglets were weighed within 24 h after birth (d 0), and then at day 7, day 14, day 21, and weaning at day 28.

### Blood sampling

Jugular blood samples were collected from all sows at 06:00 (before feeding) on day 80 and 110 of gestation, at 5 and 14 days post parturition and at weaning. Blood samples (10 mL) were collected in vacutainer tubes containing sodium heparin (Becton Dickinson and Company, Rutherford, NJ, USA). They were put on ice and centrifuged within 60 min at 4°C for 30 min at 3,000× g; serum was immediately recovered and frozen at −20°C until further analyses. Sow serum biochemical values of glucose, blood urine nitrogen (BUN), creatinine (Creat), total protein (TP), cholesterol (Chole), triglycerides (Trig), high-density lipoprotein cholesterol, low-density lipoprotein cholesterol, aspartate aminotransferase (AST), alanine aminotransferase (ALT), and alkaline phosphatase (ALK) were measured and the test methods and reagents (MeDiPro Formosa Biomedical Technology Corp., Taipei, Taiwan) that were used to analyze these parameters are shown in Table 2. The analyses for all parameters were performed at 37°C by an automated biochemical analyzer (Automatic Analyzer, HITACHI 7150. Tokyo, Japan).

### Statistical analysis

One-way analysis of variance using SPSS software [17] was used to compare the characteristics of sow serum biochemical values and performance. Comparison between treatments was done by calculating the standard error of difference between two means and least significant difference. Analysis of variance was also used to compare the characteristics of sow and piglet performance and serum biochemical parameters in sow plasma at different timepoints. The factors included in the analysis were sow dietary treatments and physiological stages. The effects of treatment on piglet performance were analyzed by analysis of variance with the litter as the experiment unit.

## RESULTS

### Sow and piglet performances

There were 54 sows throughout the whole experiment without any losses or replacements. They had an average of 113.4 ±0.21 days for pregnancy, and 3.28±0.26 hours for farrowing. The sows spent an average of 4.9±1.11 days before coming into heat after weaning, however only 49 sows (90.7%) were seen in heat in the subsequent breeding cycle.

Table 3 shows the treatment effects on body weight and backfat thickness changes. There was no difference between treatments in the initial body weight (p = 0.613). With the increase in days of pregnancy, WB and RH sows had a higher body weight gain at 80 days of pregnancy (p = 0.002). At 110 days of pregnancy, SH and RH sows had a higher body weight gain than WB sows (p<0.001). However, sows fed with different diets gained 22.0, 21.8, and 25.5 kg of net maternal body weight during gestation (for WB, SH, and RH sows, respectively; p = 0.007). There was no treatment effect on the body weight change during lactation (p = 0.158). The RH sows had lower backfat thickness at 80 days of pregnancy (p = 0.001) and at 110 days of pregnancy (p = 0.002). The WB sows had greater backfat thickness at weaning (p<0.001).

With the increase of lactation, SH and RH sows showed gradually increasing weekly feed intake, however WB sows reduced feed intake in the fourth week of lactation (Table 4). When comparing the treatments, it was seen that the sows that received soya hulls and rich hulls diets consumed more feed in the fourth week of lactation (p<0.001). The RH sows took an average of 133.66 kg of feed during lactation, while other sows took 121.29 and 126.77 kg (for WB and SH sows, respectively; p<0.001). The litter size was equalized after birth to 10 piglets, by cross-fostering from the same treatment group. The litters from SH mothers had a higher body weight at birth and gained more body weight in lactation; 11.32, 15.71, 16.24, and 16.06 kg for the 1st, 2nd, 3rd, and 4th week respectively. Overall, the SH litters gained an average of 59.34 kg of litter weight during lactation, while other litters gained 51.58 and 49.98 kg (for WB and RH litters, respectively; p<0.001).

### Biochemical parameters in sow serum

The average values of serum biochemical parameters at the five sampling points are presented in Table 5. The physiological stage at sampling had a significant influence on the concentrations of BUN, Creat, Chole, Trig, AST, and ALK. The Chole, AST, and ALK concentrations increased significantly across the physiological stages, with a lower concentration at 80 days of gestation (p = 0.004, p = 0.008, and p<0.001, respectively). Concentrations of BUN and Creat were higher at 80 days of gestation then subsequently decreased (p<0.001 and p<0.001, respectively).

The Trig concentration was the lowest at 80 days of gestation but higher at 110 days of gestation and 5 and 14 days of lactation (p = 0.045). When fed with different type of crude fiber, SH sows had lower BUN levels at 80 days of gestation (Table 6) and significantly higher levels of Trig and ALT at 110 days of gestation (Table 7). The values of serum biochemical parameters were mostly unaffected by fiber type differences during the lactation stage. However, the SH sows had higher ALT levels (p<0.001) and WB sows had lower ALK levels (p = 0.016) at 5 days of lactation (Table 8). The RH sows had higher TP levels (p = 0.001) and Trig levels (p = 0.007), but WB sows had lower ALT levels (p<0.001) and ALK levels (p = 0.007) at 14 days of lactation (Table 9). The values of TP (p = 0.03) and ALT (p = 0.045) were affected by fiber type differences at weaning (Table 10).

## DISCUSSION

### The treatment effects on sow performance

The levels of performance recorded in this experiment were similar to typical commercial performance in limited grain production areas. There were no significant differences in initial body weight. This indicates an unbiased allocation, which should be expected since animals were genetically similar and had been maintained under standard conditions until this time. In the following stages, all sows gained similar body weight but the RH sows had a higher net maternal body weight gain postpartum. These differences may be because of the different fiber consumed and utilized by the animals. Bindelle and colleagues [18] reviewed that the presence of dietary fiber lowers the apparent fecal digestibility of the crude protein and possibly the ileal digestibility, but not necessarily the efﬁciency of nitrogen retention by the animal. In this study, sows fed with soya hulls and RH tended to gain more body weight in late pregnancy. This may be caused by the pig’s adaptation to dietary fiber digestion, which is a long process that requires 5 weeks [19]; but energy digestibility is always higher with sows than growing pigs due to their greater gut transition time, consequent on their higher gastrointestinal tract volume combined to lower feed intake per unit live weight [20]. Besides the lower efﬁciency in the utilization of grain hulls energy compared to bran, the low digestibility of some fiber sources contributes to their negative impact on the density of the ration. Sows fed with RH had the worst ratio of ration DM conversion to litter weight gain at the 2nd, 3rd, and 4th weeks of lactation, and the highest DM intake during the lactation stage. These results might be because of high crude fiber content of the RH and poorer utilization of its high acid detergent fiber content. WB and SH sows raised litters with better weight gain than RH sows. This may imply that RH sows’ ration had poorer digestibility and was in agreement with Oelkle and colleagues’ results [21]. In other experiments in which the daily energy intake per sow was equalized among the gestation treatments, a greater percentage of the sows that were fed a diet containing 50% alfalfa hay completed 3 reproductive cycles, with a greater pig survival rate than control sows [7]. However, the performance of sows fed a diet containing 46% of an alfalfa-orchard grass hay mix was equal to that of control sows [22]. Additions of sugar beet pulp to the gestation diet that ranged from 25% to 50% did not improve sow performance compared with control sows when daily energy intake was equalized among treatments [3,23]. The addition of approximately 6.8% [24] or 13.35% [25] ground wheat straw as a source of fiber to a gestation diet, that was fed for respectively 5 and 3 successive parities, increased overall litter size and total litter weight at weaning compared with sows fed the control diet when basal diets intake were equalized among treatments.

In the lactation stage, sows had a similar higher body weight loss but RH sows mobilized more backfat. RH sows gained 25.5 kg maternal body weight in pregnancy, but then lost 12.8 kg body weight and lost 1.7 mm backfat thickness in lactation. The litter performance and the experimental feeding regime may explain this. There was only one type of feed for both pregnancy and lactation in each treatment and the voluntary feed intake of sows during lactation will reflect the previous feeding experience, as is well documented in lactating sows [4]. Sows chose the lactation diet spontaneously in the week preceding parturition when they were fed a fibrous gestation diet, which promotes early intake of the lactation diet [6]. However, sows fed with soya hulls need time to adapt to the dietary fiber. As can be seen, they were gaining less body weight in the first two-thirds of pregnancy.

### Biochemical parameters in sow serum

It is difficult to compare the present biochemical values with those reported in the literature because of differences in study design, type of animals, analytical techniques etc. The present biochemical values are broadly in agreement with earlier reports for gilts and sows [10,11], with the exception of AST and ALT. Our study has demonstrated that different types of fiber ingredients in the diet are important factors influencing most of the investigated reference values for sows.

The increased AST concentration from 80 day of gestation to lactation could be explained by degradation and mobilization of body reserves [11]. The ranges for AST concentrations at the individual stage level and at the neural detergent fiber percentage level were very wide, especially during the lactation stage, when compared with other published ranges [26]. Verheyen and colleagues [11] explained that increased γ-glutamyl transpeptidase concentration after farrowing might be due to liver damage, as the AST concentration showed a similar increase.

The ALK concentrations were increasing with physiological stage. However, Verheyen and colleagues [11] found a decrease during the last week of gestation and values remained at a low level after farrowing. This may be caused by the fact that the high levels of histochemical phosphatase activity in the pig placenta decrease towards the end of pregnancy and are absent after farrowing. Our finding may be due to higher levels of grain hulls’ neutral detergent fiber (NDF) and acid detergent fiber (ADF) percentage in the diet; especially SH and RH sows needed to produce more enzymes for the mobilization of body reserves and nutrient degradation to compensate for lower back fat thickness resulting from the lower density feeding regime.

The decreasing serum concentration of BUN from 80 days of gestation to lactation could be explained by higher levels of grain hulls’ NDF and ADF percentage in the diet. In growing and finishing pigs, Govers and colleagues [27] found that substrates with more fermentable resistant starch or non-starch polysaccharides maintain the microbial activity throughout the entire large intestine and decrease proteolysis occurring in the distal colon. The undigested dietary proteins and the endogenous proteins are used for synthesis of bacterial proteins, and the intense bacterial growth in the intestine enhances urea transfer from the blood to the large intestine [28]. Canh and colleagues [29] recorded a urinary: facial nitrogen ratio of 3.83 with a grain-based diet vs 1.21 with a diet containing 250 g per kg feed of sugar beet pulp.

Similar to the results of Verheyen and colleagues [11], we found a decrease in serum concentration of Creat during lactation. They explained that interpretation of the urea and Creat levels was affected by the changes in glomerular filtration rate, in food quantity and quality and in muscle mass, which may have variable effects on the serum levels of these metabolites.

The cholesterol concentrations were increasing over time. WB and RH sows tended to have significantly lower concentration of Trig in late pregnancy. This was surprising in the case of the RH diet, which used additional vegetable oil to balance the energy level. Further investigation of dietary fiber containing ingredients for sow nutrition is merited.

## CONCLUSION

During pregnancy, sows which were genetically similar and had been maintained under standard conditions gained different body weight and backfat thickness when fed various fibrous dietary ingredients. Besides the lower efficiency in the utilization of grain hulls’ energy compared to bran, the low digestibility of some fiber sources also contributed to their negative impact on the density of the ration. Sows fed with RH gained more maternal weight during gestation and consumed more feed during lactation. Additional vegetable oil was used in the RH diet to balance the energy level, however these sows still had a lower concentration of Trig in late pregnancy, indicating a need for further investigation of effects of dietary fiber content for sow nutrition. The sows fed with soya hulls had significantly better litter weight at birth and litter weight gain at weaning. Therefore, soya hulls can be a useful alternative to replace WB in sow diets. Use of locally obtained grain hull by-products in diets for sows may be an effective approach to reduce feed cost significantly, whilst maintaining the serum biochemical parameters at the average level and increasing voluntary feed intake in lactation.

## Figures and Tables

**Table 1:** Ingredient composition (%) of gestation and lactation diets1)

Items	WB2)	SH3)	RH4)
Ingredient (%)			
Yellow corn	47.8	45.3	41.3
Soybean seed dehulled, 42.4% CP	6.5	10	12.5
Soybean meal dehulled, 53.5% CP	12	11	14
Barley	10	10	6
Wheat bran	20	-	-
Soya hulls	-	20	-
Rice hulls	-	-	20
Dicalcium phosphate	1.2	1.2	1.2
Limestone, pulverized	1.7	1.7	1.7
Soya oil	-	-	2.5
Salt	0.3	0.3	0.3
Choline chloride (50%)	0.1	0.1	0.1
Vitamin premix5)	0.2	0.2	0.2
Mineral premix5)	0.2	0.2	0.2
Calculated composition (% as fed)			
Dry matter	86.86	87.42	88.43
Crude protein	16.02	16.03	16.02
Crude fiber	4.08	9.22	10.06
Ether extract	4.11	4.37	6.99
Neutral detergent fiber	16.97	20.49	20.75
Acid detergent fiber	4.92	10.86	12.08
Ash	2.87	2.90	5.37
Calcium	0.96	1.03	0.96
Total phosphorus	0.69	0.53	0.54
Lysine (g/kg as fed)	7.94	8.71	8.55
Methionine (g/kg as fed)	2.61	2.54	2.58
Cystine (g/kg as fed)	3.19	3.16	3.06
DE (Kcal/kg as fed)	3,570	3,529	3,447
ME (Kcal/kg as fed)	3,424	3,379	3,288
Daily ration intake (kg)			
1 to 80 days of gestation	2.02	2.05	2.10
81 to 112 days of gestation	2.39	2.42	2.48
113 days of gestation to lactation	2.0	2.0	2.0
Lactation period	NR6)	NR6)	NR6)

CP, crude protein; DE, digestible energy; ME, metabolisable energy.

1) The rations were fed to sows in gestation and lactation in each treatment.

2) 20% as fed of wheat bran supplementation.

3) 20% as fed of soya hulls supplementation.

4) 20% as fed of rice hulls supplementation.

5) Vitamin and trace mineral premix (China Chemical & Pharmaceutical Co., Ltd., Taiwan) provided, per kilogram of control diet or diet containing corn silage: 6,600 IU of vitamin A acetate; 1,210 IU of vitamin D_3_; 22.0 IU of vitamin E from dl-α-tocopheryl acetate; 3.5 mg of vitamin K from menadione sodium dimethylprimidinol bisulfite; 22.0 mg of pantothenic acid as d-calcium pantothenate; 33.2 mg of niacin; 2.0 mg of folic acid; 5.5 mg of riboflavin; 27.6 μg of vitamin B_12_; 330 μg of biotin from d-biotin; 575 mg of choline from choline chloride; 125 mg of Zn as ZnSO_4_; 126 mg of Fe as FeSO_4_; 60 mg of Mn as MnSO_4_; 0.55 mg of I as Ca(IO_3_)_2_; and 0.35 mg of Se as Na_2_SeO_3_.

6) The feed was increased postpartum by 0.5 kg DM per day till sows reached ad libitum intake.

**Table 2:** The units and methods used for the biochemical analyses

Variables	Methods	Number1)
Glucose (mg/dL)	Enzymatic colometric test	BC-0019
Blood urine nitrogen (mg/dL)	Bromcresol green method	BC-0012
Creatinine (mg/dL)	Picric acid test	BC-0017
Total protein (g/dL)	Biuret method	BC-0026
Cholesterol (mg/dL)	Enzymatic method	BC-0014
Triglycerides (mg/dL)	Enzymatic method	BC-0027
High-density lipoprotein cholesterol (mg/dL)	Direct method	BC-0020
Low-density lipoprotein cholesterol (mg/dL)	Direct method	BC-0023
Aspartate aminotransferase (U/L)	UV-IFCC method	BC-0008
Alanine aminotransferase (U/L)	UV-IFCC method	BC-0007
Alkaline phosphatase (U/L)	IFCC method	BC-0006

1) MeDiPro Formosa Biomedical Technology Corp., Taipei, Taiwan.

**Table 3:** The effects of dietary supplementation with different types of fiber in gestation and lactation on sow body weight and backfat thickness changes

Items	WB1)	SH2)	RH3)	SEM	p-value
Initial body weight at breeding (kg)	200.6	203.3	201.0	7.11	0.613
Body weight changes to 80 days of pregnancy (kg)	21.3^a^	18.8^b^	21.3^a^	2.15	0.002
Body weight changes to 110 days of pregnancy (kg)	16.0^b^	20.5^a^	20.5^a^	3.13	<0.001
Net pregnancy maternal body weight gain (kg)4)	22.0^b^	21.8^b^	25.5^a^	3.40	0.007
Litter weight at birth (kg)	15.3	17.4	16.4	2.40	0.087
Body weight changes to weaning (kg)	14.2	14.2	12.8	2.11	0.158
Backfat thickness at 30 days of pregnancy (mm)	15.2	14.6	14.5	1.21	0.091
Backfat thickness at 80 days of pregnancy (mm)	16.1^a^	15.3^a^	14.8^b^	1.32	0.001
Backfat thickness at 110 days of pregnancy (mm)	18.1^a^	16.8^a^	15.2^b^	2.16	0.002
Backfat thickness at weaning (mm)	17.8^a^	14.9^b^	13.5^b^	2.52	<0.001

SEM, standard error of the mean.

1) 20% as fed of wheat bran supplementation.

2) 20% as fed of soya hulls supplementation.

3) 20% as fed of rice hulls supplementation.

4) Net maternal body weight gain during pregnancy.

a,b Means within a row without a common superscript letter differ (p<0.05).

**Table 4:** The effects of dietary supplementation with different types of fiber in gestation and lactation on sow performance during lactation

Items	WB1)	SH2)	RH3)	SEM	p-value
Total dry matter intake in 1st week (kg)	25.64^b^	28.58^a^	29.62^a^	0.423	0.002
Total dry matter intake in 2nd week (kg)	31.20	30.77	32.41	0.501	0.387
Total dry matter intake in 3rd week (kg)	32.64	32.76	34.52	0.391	0.097
Total dry matter intake in 4th week (kg)	31.80^c^	34.67^b^	37.11^a^	0.446	<0.001
Total dry matter intake in lactation (kg)	121.29^c^	126.77^b^	133.66^a^	0.932	<0.001
Litter weight at birth (kg)4)	15.24^b^	17.62^a^	15.49^b^	0.113	<0.001
Litter weight gain in 1st week (kg)	10.98^ab^	11.32^a^	10.60^b^	0.087	0.007
Litter weight gain in 2nd week (kg)	13.70^b^	15.71^a^	12.29^c^	0.162	<0.001
Litter weight gain in 3rd week (kg)	14.57^b^	16.24^a^	13.63^c^	0.104	<0.001
Litter weight gain in 4th week (kg)	12.32^b^	16.06^a^	13.46^c^	0.082	<0.001
Total litter weight gain in lactation (kg)	51.58^b^	59.34^a^	49.98^c^	0.216	<0.001

SEM, standard error of the mean.

1) 20% as fed of wheat bran supplementation.

2) 20% as fed of soya hulls supplementation.

3) 20% as fed of rice hulls supplementation.

4) The litter size was equalized to 10 piglets from the same treatment at 2 to 3 days old.

a–c Means within a row without a common superscript letter differ (p<0.05).

**Table 5:** The effect of physiological stage on serum biochemical values

Items	80-D-G1)	110-D-G2)	5-D-L3)	14-D-L4)	28-D-L5)	SEM	p-value	Reference6)
GLU (mg/dL)	72.9	76.4	78.9	73.4	73.9	1.359	0.602	85–150
BUN (mg/dL)	18.8^a^	14.1^b^	15.5^b^	14.7^b^	15.3^b^	0.321	<0.0001	10–30
Creat (mg/dL)	1.19^a^	1.01^b^	1.03^b^	1.01^b^	0.96^b^	0.013	<0.0001	1.0–2.7
TP (g/dL)	7.33	7.29	7.70	7.48	7.37	0.053	0.121	7.9–8.9
Chole (mg/dL)	60.6^b^	72.3^a^	74.5^a^	72.9^a^	69.6^a^	1.201	0.004	51–111*
Trig (mg/dL)	42.0^b^	55.0^a^	54.3^a^	58.5^a^	48.5^ab^	1.814	0.045	15–75*
HDL-C (mg/dL)	29.9	35.0	35.8	34.1	33.2	0.767	0.143	12–35*
LDL-C (mg/dL)	22.3	26.3	27.9	27.6	26.7	0.749	0.142	26–79*
AST (U/L)	70.8^b^	85.4^a^	90.2^a^	85.9^a^	86.3^a^	1.724	0.008	32–84
ALT (U/L)	57.3	52.1	56.1	59.4	54.8	1.535	0.636	31–58
ALK (U/L)	77.0^b^	120.7^a^	134.2^a^	130.6^a^	134.9^a^	2.969	<0.0001	118–395

SEM, standard error of the mean; GLU, glucose; BUN, blood urine nitrogen; Creat, creatinine; TP, total protein; Chole, cholesterol; Trig, triglycerides; HDL-C, high-density lipoprotein cholesterol; LDL-C, low-density lipoprotein cholesterol; AST, aspartate aminotransferase; ALT, alanine aminotransferase; ALK, alkaline phosphatase.

1) 80 days of gestation.

2) 110 days of gestation.

3) 5 days of lactation.

4) 14 days of lactation.

5) 28 days of lactation.

6) Latimer et al [30].

* Uddin et al [31].

a–b Means within a row without a common superscript letter differ (p<0.05).

**Table 6:** The effect of dietary supplementation with different types of fiber on serum biochemical values for 54 sows at 80 days of gestation

Items	WB1)	SH2)	RH3)	SEM	p-value
GLU (mg/dL)	75.8	64.8	79.8	4.089	0.311
BUN (mg/dL)	20.3^a^	16.7^b^	19.8^a^	0.426	0.007
Creat (mg/dL)	1.20	1.26	1.11	0.036	0.269
TP (g/dL)	7.26	7.36	7.37	0.128	0.931
Chole (mg/dL)	57.9	61.5	62.3	1.984	0.650
Trig (mg/dL)	40.8	41.0	44.3	2.852	0.859
HDL-C (mg/dL)	29.2	30.8	29.5	1.137	0.816
LDL-C (mg/dL)	20.5	22.5	23.9	1.737	0.743
AST (U/L)	63.1	71.4	77.8	4.026	0.376
ALT (U/L)	53.3	64.8	52.3	3.915	0.356
ALK (U/L)	73.5	82.2	74.3	4.648	0.694

SEM, standard error of the mean; GLU, glucose; BUN, blood urine nitrogen; Creat, creatinine; TP, total protein; Chole, cholesterol; Trig, triglycerides; HDL-C, high-density lipoprotein cholesterol; LDL-C, low-density lipoprotein cholesterol; AST, aspartate aminotransferase; ALT, alanine aminotransferase; ALK, alkaline phosphatase.

1) 20% as fed of wheat bran supplementation.

2) 20% as fed of soya hulls supplementation.

3) 20% as fed of rice hulls supplementation.

a,b Means within a row without a common superscript letter differ (p<0.05).

**Table 7:** The effect of dietary supplementation with different types of fiber on serum biochemical values for 54 sows at 110 days of gestation

Items	WB1)	SH2)	RH3)	SEM	p-value
GLU (mg/dL)	68.3	82.5	77.3	3.107	0.202
BUN (mg/dL)	14.3	13.6	14.6	0.986	0.907
Creat (mg/dL)	1.01	1.05	0.95	0.031	0.453
TP (g/dL)	7.44	7.19	7.27	0.134	0.745
Chole (mg/dL)	73.8	75.3	67.3	4.353	0.733
Trig (mg/dL)	44.6^b^	76.9^a^	39.1^b^	5.161	0.018
HDL-C (mg/dL)	36.1	36.6	32.0	2.833	0.776
LDL-C (mg/dL)	28.8	23.4	27.5	2.511	0.650
AST (U/L)	74.9	94.0	85.7	5.476	0.379
ALT (U/L)	41.5^b^	64.1^a^	48.3^ab^	2.996	0.021
ALK (U/L)	108.8	134.8	115.8	9.627	0.522

SEM, standard error of the mean; GLU, glucose; BUN, blood urine nitrogen; Creat, creatinine; TP, total protein; Chole, cholesterol; Trig, triglycerides; HDL-C, high-density lipoprotein cholesterol; LDL-C, low-density lipoprotein cholesterol; AST, aspartate aminotransferase; ALT, alanine aminotransferase; ALK, alkaline phosphatase.

1) 20% as fed of wheat bran supplementation.

2) 20% as fed of soya hulls supplementation.

3) 20% as fed of rice hulls supplementation.

a,b Means within a row without a common superscript letter differ (p<0.05).

**Table 8:** The effect of dietary supplementation with different types of fiber on serum biochemical values for 54 sows at 5 days of lactation

Items	WB1)	SH2)	RH3)	SEM	p-value
GLU (mg/dL)	77.1	79.0	80.6	2.217	0.817
BUN (mg/dL)	16.0	15.4	15.1	0.719	0.892
Creat (mg/dL)	1.03	1.05	1.02	0.019	0.696
TP (g/dL)	7.78	7.46	7.91	0.080	0.089
Chole (mg/dL)	75.3	76.0	72.0	2.353	0.770
Trig (mg/dL)	46.5	63.8	50.9	2.815	0.057
HDL-C (mg/dL)	36.6	37.0	33.6	1.600	0.651
LDL-C (mg/dL)	29.4	26.2	28.3	1.259	0.579
AST (U/L)	81.8	91.6	96.8	2.562	0.098
ALT (U/L)	45.7^b^	67.5^a^	53.0^b^	1.689	<0.0001
ALK (U/L)	114.6^b^	145.6^a^	140.1^a^	3.956	0.016

SEM, standard error of the mean; GLU, glucose; BUN, blood urine nitrogen; Creat, creatinine; TP, total protein; Chole, cholesterol; Trig, triglycerides; HDL-C, high-density lipoprotein cholesterol; LDL-C, low-density lipoprotein cholesterol; AST, aspartate aminotransferase; ALT, alanine aminotransferase; ALK, alkaline phosphatase.

1) 20% as fed of wheat bran supplementation.

2) 20% as fed of soya hulls supplementation.

3) 20% as fed of rice hulls supplementation.

a,b Means within a row without a common superscript letter differ (p<0.05).

**Table 9:** The effect of dietary supplementation with different types of fiber on serum biochemical values for 54 sows at 14 days of lactation

Items	WB1)	SH2)	RH3)	SEM	p-value
GLU (mg/dL)	73.4	75.3	71.2	2.111	0.739
BUN (mg/dL)	15.2	14.6	14.4	0.684	0.892
Creat (mg/dL)	0.98	1.00	1.04	0.018	0.481
TP (g/dL)	7.41^b^	7.10^b^	8.01^a^	0.077	0.001
Chole (mg/dL)	71.7	72.4	74.8	2.241	0.843
Trig (mg/dL)	44.6^b^	60.8^ab^	70.1^a^	2.681	0.007
HDL-C (mg/dL)	34.9	35.3	31.9	1.524	0.637
LDL-C (mg/dL)	28.0	25.0	30.4	1.199	0.211
AST (U/L)	77.9	87.2	92.3	2.440	0.096
ALT (U/L)	43.5^b^	64.3^a^	69.6^a^	1.609	<0.0001
ALK (U/L)	109.1^b^	138.7^a^	142.3^a^	3.768	0.007

SEM, standard error of the mean; GLU, glucose; BUN, blood urine nitrogen; Creat, creatinine; TP, total protein; Chole, cholesterol; Trig, triglycerides; HDL-C, high-density lipoprotein cholesterol; LDL-C, low-density lipoprotein cholesterol; AST, aspartate aminotransferase; ALT, alanine Aminotransferase; ALK, alkaline phosphatase.

1) 20% as fed of wheat bran supplementation.

2) 20% as fed of soya hulls supplementation.

3) 20% as fed of rice hulls supplementation.

a,b Means within a row without a common superscript letter differ (p<0.05).

**Table 10:** The effect of dietary supplementation with different types of fiber on serum biochemical values for 54 sows at 28 days of lactation

Items	WB1)	SH2)	RH3)	SEM	p-value
GLU (mg/dL)	78.5	68.0	76.3	3.153	0.366
BUN (mg/dL)	16.1	15.7	14.2	0.747	0.582
Creat (mg/dL)	0.95	0.96	0.98	0.043	0.959
TP (g/dL)	7.37^ab^	7.01^b^	7.79^a^	0.106	0.030
Chole (mg/dL)	69.6	69.4	69.9	2.623	0.997
Trig (mg/dL)	43.9	44.6	57.8	2.674	0.099
HDL-C (mg/dL)	33.6	33.9	32.0	1.466	0.852
LDL-C (mg/dL)	27.2	26.6	26.3	1.690	0.977
AST (U/L)	80.9	80.4	98.7	3.063	0.051
ALT (U/L)	45.5^b^	64.4^a^	52.6^ab^	2.833	0.045
ALK (U/L)	109.4	142.6	151.1	6.849	0.069

SEM, standard error of the mean; GLU, glucose; BUN, blood urine nitrogen; Creat, creatinine; TP, total protein; Chole, cholesterol; Trig, triglycerides; HDL-C, high-density lipoprotein cholesterol; LDL-C, low-density lipoprotein cholesterol; AST, aspartate aminotransferase; ALT, alanine Aminotransferase; ALK, alkaline phosphatase.

1) 20% as fed of wheat bran supplementation.

2) 20% as fed of soya hulls supplementation.

3) 20% as fed of rice hulls supplementation.

a,b Means within a row without a common superscript letter differ (p<0.05).
